# Maternal and Fetal Outcomes of Pregnancy-related Hypertensive Disorders in a Tertiary Care Hospital in Sukkur, Pakistan

**DOI:** 10.7759/cureus.5507

**Published:** 2019-08-28

**Authors:** Shoaib Un Nisa, Altaf A Shaikh, Raj Kumar

**Affiliations:** 1 Obstetrics and Gynecology, Ghulam Muhammad Mahar Medical College and Hospital, Sukkur, PAK; 2 Internal Medicine, Ghulam Muhammad Mahar Medical College and Hospital, Sukkur, PAK; 3 Cardiology, Ghulam Muhammad Mahar Medical College and Hospital, Sukkur, PAK

**Keywords:** maternal complications, fetal complication, hypertensive disorder of pregnancy, eclampsia

## Abstract

Introduction

Pregnancy-related hypertensive disorders are a significant cause of adverse maternal and fetal outcomes, especially in developing areas of the world. Preeclampsia and eclampsia are the most significant causes of maternal and perinatal morbidity and mortality.

Methods

We conducted a prospective observational study in the Obstetrics and Gynaecology Department of Ghulam Muhammad Meher Medical College and Hospital. One hundred twelve (n=112) women with pregnancy-related hypertensive disorders admitted for delivery were included in the study.

Results

The major pregnancy-related hypertensive disorder was eclampsia (n=48; 43.24%) and preeclampsia (n=28; 25.23%). Among the women who developed one or more complications during or after delivery, postpartum hemorrhage (PPH) was the most frequent (n=31; 27.6%).

Conclusions

Pregnancy-related hypertensive disorders are common and adversely impact maternal and fetal outcomes. Efforts should be made at both the community and hospital levels to increase awareness regarding hypertensive disorder of pregnancy and reduce its associated morbidity and mortality.

## Introduction

Every 60 minutes, three women die in Pakistan due to pregnancy- and childbirth-related complications. Pregnancy-related hypertensive disorders are prevalent in 6% to 8% of all pregnancies [1]. They are considered a significant cause of adverse maternal and fetal outcomes globally, more so in developing areas of the world [2]. Hypertensive disorders of pregnancy include a spectrum of diseases ranging from mildly elevated blood pressure to multi-organ dysfunction [3]. Organizations such as the American College of Obstetricians and Gynecologists (ACOG) and the United Nations Organization have classified pregnancy-related hypertensive disorders into four categories: chronic hypertension (HTN), pregnancy-induced hypertension (PIH), preeclampsia/eclampsia, and superimposed preeclampsia/eclampsia [4-5]. HTN with proteinuria (diagnosed as preeclampsia) normally occurs after the 20th week of gestation and is responsible for complicating 2% to 8% of pregnancies [6]. Pregnancy-related hypertensive emergencies may present as HELLP (hemolysis, elevated liver enzymes, low platelets) syndrome or eclampsia (preeclampsia with seizures) [3]. After hemorrhage, preeclampsia and eclampsia are considered the most significant causes of maternal and perinatal mortality and morbidity [3,7].

Global literature has identified various risk factors for hypertensive disorders in pregnancy, such as obesity, family history of HTN, alcohol use, heart failure, stroke, smoking, and left ventricular hypertrophy [8-10]. When these high-risk pregnancies are followed until childbirth, up to 22% of pregnancies resulted in maternal and fetal complications [11]. The risk of adverse events in newborns depends on the severity of hypertensive disorders during pregnancy; for example, the risk of preterm delivery was minimum in normotensive mothers (7.2%), higher in mothers with PIH (12.5%), and highest in women with preeclampsia (39.2%) [12]. Although globally a lot of work has been done on this topic, there are not enough data available from Pakistan, especially in rural areas. In this study, we assess maternal and fetal outcomes in mothers with hypertensive disorder of pregnancy from Sukkur. The result from this article will help us highlight the need for proper awareness campaign regarding hypertensive disorder in pregnancy.

## Materials and methods

A prospective observational study was conducted in the Obstetrics and Gynaecology Department of Ghulam Muhammad Mahar Medical College and Hospital from January 1 to December 31, 2018. The study was approved by the institutional review board, and written informed consent was attained from all patients. Eligible patients included those diagnosed with at least one pregnancy-related hypertensive disorder (chronic HTN, chronic HTN superimposed, PIH, preeclampsia, eclampsia, and HELLP syndrome) who were admitted in the department for delivery/expulsion. Hypertensive disorder of pregnancy was diagnosed per ACOG definition [13]. Patients with no pregnancy-related hypertensive disorder, who did not consent to participate, or those who were received in such an emergency that they were in delirium and were unable to communicate were excluded from the study.

Patient age, gestational age confirmed via ultrasound, blood pressure, and type of pregnancy-related hypertensive disorder were recorded for all participants. All patients were observed for the development of any complication during their hospital stay (before, during, and after delivery/expulsion). The maternal outcome was recorded, and the incidence of any neonatal complication was observed.

Data were analyzed using IBM SPSS Statistics for Windows, Version 22.0. (IBM Corp., Armonk, NY). The mean and standard deviation was calculated for numerical values such as age and blood pressure at the time of admission. Frequencies were calculated for hypertensive disorders of pregnancy, as well as maternal and fetal complications.

## Results

There were 2,012 deliveries conducted in the hospital during the study period. Out of these, 112 (5.56%) were diagnosed cases of pregnancy-related hypertensive disorders, which were included in the study. The mean age of participants was 23 ± 5 years. Mean systolic pressure was 160.95 ± 13.86 mmHg, mean diastolic pressure was 103.68 ± 6.291 mmHg, and mean gestational age was 35.95 ± 2.849 weeks. The frequency of various hypertensive disorders in our sample is shown in Table 1.

**Table 1:** Frequencies of hypertensive disorders HELLP, hemolysis, elevated liver enzymes, and low platelets.

Hypertensive Disorder	Frequency (%)
Pregnancy-induced hypertension	26 (23.2%)
Preeclampsia	28 (25.0%)
Eclampsia	48 (42.8%)
Chronic hypertension	5 (4.5%)
Chronic hypertension superimposed	3 (2.7%)
HELLP	2 (1.8%)
Total	112 (100%)

All of these women were observed for the development of any complication. There were no complications in 38 (33.9%) women. Among the women who developed one or more complications during or after delivery, postpartum hemorrhage (PPH) was the most frequent (31/112; 27.6%). Most of the women with PPH were either preeclamptic or eclamptic. After PPH, placental abruption was the second most frequent maternal complication (19/112; 16.9%). Most of the women who developed placental abruption had PIH. All details of maternal complications are shown in Table 2.

**Table 2:** Trends of maternal complications ARDS, acute respiratory distress syndrome; ARF, acute renal failure; CVA, cerebrovascular accident; DIC, disseminated intravascular coagulation; HELLP, hemolysis elevated liver enzymes low platelets; HTN, hypertension; P. edema, pulmonary edema; PIH, pregnancy-induced hypertension; PPH, postpartum hemorrhage; PRES, posterior reversible encephalopathy syndrome.

Pregnancy-related HTN disorders (N=112)	Maternal Complications								
	Placental abruption	DIC	PPH	ARF	ARDS	PRES	P. edema	CVA	No complication
PIH (n=26; 23.2%)	9 (47.3%)	0	9 (29%)	0	0	0	2 (28.5%)	0	6 (15.8%)
Preeclampsia (n=28; 25%)	6 (31.6%)	2 (40%)	9 (29%)	2 (28.5%)	2 (50%)	0	1 (14.3%)	0	6 (15.8%)
Eclampsia (n=48; 42.8%)	3 (15.8%)	0	11 (35.5%)	3 (42.8%)	1 (25%)	2 (100%)	4 (57.2%)	3 (100%)	21 (55.3%)
HELLP (n=2; 1.8%)	0	2 (40%)	0	1 (14.3%)	1 (25%)	0	0	0	0 (%)
Chronic HTN (n=5; 4.5%)	0	0	0	0	0	0	0	0	5 (13.2%)
Chronic HTN superimposed (n=3; 2.7%)	1 (5.2%)	1 (20%)	2 (6.5%)	1 (14.3%)	0	0	0	0	0
Total (N=112; 100%)	19 (100%)	5 (100%)	31 (100%)	7 (100%)	4 (100%)	2 (100%)	7 (100%)	3 (100%)	38 (100%)

When the neonatal outcome was evaluated, 11 intrauterine deaths, four stillbirths, and two neonatal deaths were observed. There were no multiple pregnancies in the study sample. When the remaining 95 alive newborns were examined, 44 were alert and healthy with no immediate neonatal complication. Among the remaining 51 newborns with complications, 14 babies were diagnosed with meconium aspiration syndrome, 12 were preterm, 10 had low birth weight (LBW), 10 had intrauterine growth restriction (IUGR), and five were diagnosed with respiratory distress syndrome (RDS). No complications were seen in the babies of mothers with chronic HTN and chronic HTN superimposed. All details of neonatal outcomes are shown in Table 3.

**Table 3:** Trends of neonatal complications HELLP, hemolysis elevated liver enzymes low platelets; HTN, hypertension; IUD, intrauterine death; IUGR, intrauterine growth restriction; LBW, low birth weight; NND, neonatal death; PIH, pregnancy-induced hypertension; RDS, respiratory distress syndrome.

Pregnancy-related HTN disorders (N=112)	Neonatal Complications								
	IUGR	Preterm	RDS	Meconium aspiration	IUD	Stillbirth	LBW	NND	No complication
PIH (n=26; 23.2%)	1 (10%)	0	1 (20%)	4 (28.5%)	3 (27.3%)	0	3 (30%)	2 (100%)	12 (27.3%)
Preeclampsia (n=28; 25%)	1 (10%)	3 (25%)	3 (60%)	3 (21.4%)	2 (18.2%)	0	5 (50%)	0	11 (25%)
Eclampsia (n=48; 42.8%)	8 (80%)	8 (66.7%)	1 (20%)	7 (50%)	5 (45.5%)	4 (100%)	2 (20%)	0	13 (29.5%)
HELLP (n=2; 1.7%)	0	1 (8.3%)	0	0	1 (9 %)	0	0	0	0
Chronic HTN (n=5; 4.5%)	0	0	0	0	0	0	0	0	5 (11.4%)
Chronic HTN superimposed (n=3; 2.7%)	0	0	0	0	0	0	0	0	3 (6.8%)
Total (N=112; 100%)	10 (100%)	12 (100%)	5 (100%)	14 (100%)	11 (100%)	4 (100%)	10 (100%)	2 (100%)	44 (100%)

## Discussion

Currently, Pakistan is ranked third in terms of the burden of maternal, fetal, and child mortality [14]. According to a study published in 2007, pregnancy-related disorder (eclampsia) was responsible for one-third of maternal deaths in women admitted for delivery in a tertiary care hospital in Pakistan. In this study, 5.56% of pregnant women were diagnosed with pregnancy-related hypertensive disorders; the major contributors were eclampsia (43.24%) and preeclampsia (25.23%) [14]. A report published by the American Journal of Obstetrics and Gynecology stated that 4% to 6% of pregnancies are complicated by hypertensive disorders [1]. Another study indicated that the incidence of pregnancy-related hypertensive disorders may be as high as up to 22% [15]. Various reasons have been identified for the high prevalence of eclampsia and preeclampsia in the local literature, including lack of education, awareness, resources, and superstitious beliefs regarding seeking medical aid [16]. Another major challenge is a delay in diagnosis and recognition of preeclampsia and eclampsia, which accounts for high maternal complications as well as mortality, which is otherwise preventable [17-18].

Preeclampsia and eclampsia are the major causes of high morbidity and mortality for both mother and baby, particularly in developing countries [19]. Placental abruption and PPH were the most common maternal complications in pregnant women with preeclampsia and eclampsia in this study. Other complications experienced were disseminated intravascular coagulation, acute renal failure, acute RDS, posterior reversible encephalopathy syndrome, and pulmonary edema. This result was comparable to the results of studies conducted in India, which also reported placental abruption as a major concern [20].

Pregnancy-related hypertensive disorders are not only a concern for maternal health but also fetal health. The most common neonatal complication seen in preeclampsia in this study was LBW. In his study, Xiong et al. found a positive association not only between LBW and preeclampsia but also between gestational age and preeclampsia [21]. The most common fetal complications in pregnant women with eclampsia in this study were IUGR and preterm birth. A study published in Tehran also identified eclampsia as a risk factor for preterm birth [22]. In this study, there were four stillbirths, all in eclamptic women.

This study had its limitations. First, both exposure (hypertensive disorders of pregnancy) and outcome (maternal and fetal complications) were measured at the same time. Second, it was a single-center study with a small sample, so the results cannot be generalized. Also, since the sampling technique used was convenient consecutive sampling, selection bias might have been introduced.

## Conclusions

The incidence of hypertensive disorders of pregnancy is 5.56% in a tertiary care hospital in Sukkur, Pakistan. The most prevalent hypertensive disorders were preeclampsia and eclampsia. The most common associated maternal complications were placental abruption and PPH. The most common associated fetal complications were meconium aspiration syndrome, followed by preterm birth, IUGR, and LBW. Efforts should be made to reduce the risk factors responsible for the high incidence of preeclampsia and eclampsia at the grass-roots level. Awareness and resources should be made available at all levels to reduce the maternal and fetal complications associated with hypertensive disorders of pregnancy. Programs should be introduced to raise awareness at the community level, and health facilities should be well equipped to make early detection and manage preeclampsia and other hypertensive disorders adequately.
